# Indoor Environmental Exposures and Dry Eye Disease: Mechanisms, Clinical Impact, and Prevention Strategies

**DOI:** 10.3390/toxics14080711

**Published:** 2026-08-12

**Authors:** Yumeng Li, Ji Yang, Tao Xie, Ping Xiang, Lei Kong, Hai Liu

**Affiliations:** 1 Department of Ophthalmology, The Eye Disease Clinical Medical Research Center of Yunnan Province, The Eye Disease Clinical Medical Center of Yunnan Province, The Second People’s Hospital of Yunnan Province, The Affiliated Hospital of Yunnan University, Kunming 650021, China; liyumeng_927@163.com (Y.L.); yangji@ynu.edu.cn (J.Y.); 13888312791@163.com (T.X.); 2 Institute of Environmental Remediation and Human Health, School of Ecology and Environment, Southwest Forestry University, Kunming 650233, China; xiangping@swfu.edu.cn

**Keywords:** indoor environmental exposures, dry eye syndrome, ocular surface, tear layer, oxidative damage, indoor environmental exposure factor

## Abstract

Dry eye disease (DED) is a complex ocular surface disorder characterized by tear film instability, discomfort, hyperosmolarity, inflammation, and neurosensory dysfunction. Since individuals spend a significant amount of time inside buildings, exposure to indoor environments has emerged as a noteworthy and adjustable factor influencing ocular surface disorders. This analysis consolidates recent findings connecting indoor air contaminants, such as particulate pollutants, volatile organic substances, formaldehyde, and other gaseous irritants, as well as tobacco and cooking fumes, heavy metals, organophosphate flame retardants, and liquid crystal compounds, with the mechanisms and symptoms associated with DED. Research suggests that indoor environmental factors can exacerbate DED by triggering interconnected mechanisms such as oxidative damage, inflammation, lipid degradation, meibomian gland issues, decreased tear production, impairment of goblet cells and the mucin layer, disruption of tight junctions, and damage to the corneal or conjunctival epithelium. The most robust clinical evidence currently pertains to tobacco smoke, particulate matter, indoor air pollution, low humidity, and poor ventilation. In contrast, although biological plausibility exists for heavy metals, flame retardants, and liquid crystal monomers, the supporting data remain relatively sparse, indirect, and less comprehensive. Harmonized exposure assessments, prospective cohort studies, analyses of pollutant mixtures, and experimental intervention trials are needed to better characterize dose–response relationships and to establish effective preventive measures. Assessment of environmental history and improvement of indoor air quality should be regarded as integral and interrelated components in the prevention and management of DED.

## 1. Introduction

### 1.1. Definition, Epidemiology, and Pathological Framework of Dry Eye Disease

Dry eye disease (DED) ranks among the most common conditions affecting the ocular surface, leading to eye irritation, fluctuating vision, decreased workplace efficiency, and a diminished overall quality of life. According to the TFOS DEWS III Diagnostic Methodology Report, dry eye is defined as “a multifactorial, symptomatic disease characterized by a loss of homeostasis of the tear film and/or ocular surface, in which tear film instability and hyperosmolarity, ocular surface inflammation and damage, and neurosensory abnormalities are etiological factors” [1]. The disease spectrum includes both aqueous-deficient and evaporative subtypes, which frequently overlap in clinical presentation and underlying mechanisms [2]. Meibomian gland dysfunction (MGD), instability of the tear film lipid layer, mucin deficiency, and compromise of the epithelial barrier are key elements within this pathological framework [3,4].

### 1.2. The Association Between Indoor Environmental Exposure and DED

Exposure to environmental factors is now widely recognized as a modifiable influence on ocular surface health [5]. Outdoor air pollution, meteorological patterns, personal habits, and indoor air quality have all been linked to DED symptoms and signs in various epidemiological, clinical, and mechanistic studies [6]. Indoor exposure is particularly relevant because contemporary populations spend a substantial amount of time inside homes, offices, educational institutions, vehicles, medical centers, and other confined spaces. Indoor air quality is influenced by outdoor air infiltration, building materials, furnishings, electronic devices, household cleaning products, cooking practices, tobacco use, ventilation systems, ambient temperature, and humidity levels [7].

### 1.3. The Complexity of Indoor Environmental Exposures

This review focuses on a broad range of indoor environmental exposures, encompassing chemical contaminants, as well as physical factors such as humidity, ventilation, and mask-related airflow. Exposure can also occur through settled dust, skin contact, or absorption into the body. This applies to many of the pollutants that we discuss: heavy metals, flame retardants, and liquid crystal monomers (LCMs). The evidence is not equally strong for all pollutant types. For factors like tobacco smoke, particulate matter, volatile organic compounds (VOCs), indoor humidity, and ventilation, there is more direct evidence linking them to DED. For heavy metals, flame retardants, and LCMs, the evidence is mostly from biomarker studies, occupational research, and reports investigating toxicology and possible biological pathways.

This review brings together recent findings on how indoor air contaminants relate to the mechanisms and symptoms of DED. The key focuses are assessing the strength of the evidence and identifying where knowledge is still lacking.

### 1.4. Literature Search Strategy and Evidence Synthesis

A literature search was conducted in PubMed and China National Knowledge Infrastructure (CNKI) databases for publications published up to April 2026. Search terms covered DED-related terms (“dry eye disease”, “ocular surface”, and “tear film”) and indoor exposure terms (“indoor air pollution”, “particulate matter”, “VOCs”, “formaldehyde”, “tobacco smoke”, “heavy metals”, “flame retardants”, “liquid crystal monomers”, and “humidity”). Only English- and Chinese-language articles were included. Priority was given to systematic reviews, meta-analyses, epidemiological studies, and mechanistic investigations published within the past five years, while foundational and highly relevant studies from earlier periods were also included when they provided essential mechanistic insights or quantitative data not available in more recent literature. Foundational reports, including TFOS DEWS II and III, were also included where essential to define key concepts. The present work was conducted as a comprehensive narrative review rather than a structured systematic review or meta-analysis, and it followed the framework of the TFOS DEWS III reports, including their executive summary [8]. Publications were reviewed if they addressed DED mechanisms, tear film biology, ocular surface inflammation, indoor air quality, indoor environmental exposure, or toxicological processes relevant to ocular surface damage.

**Figure 1:**
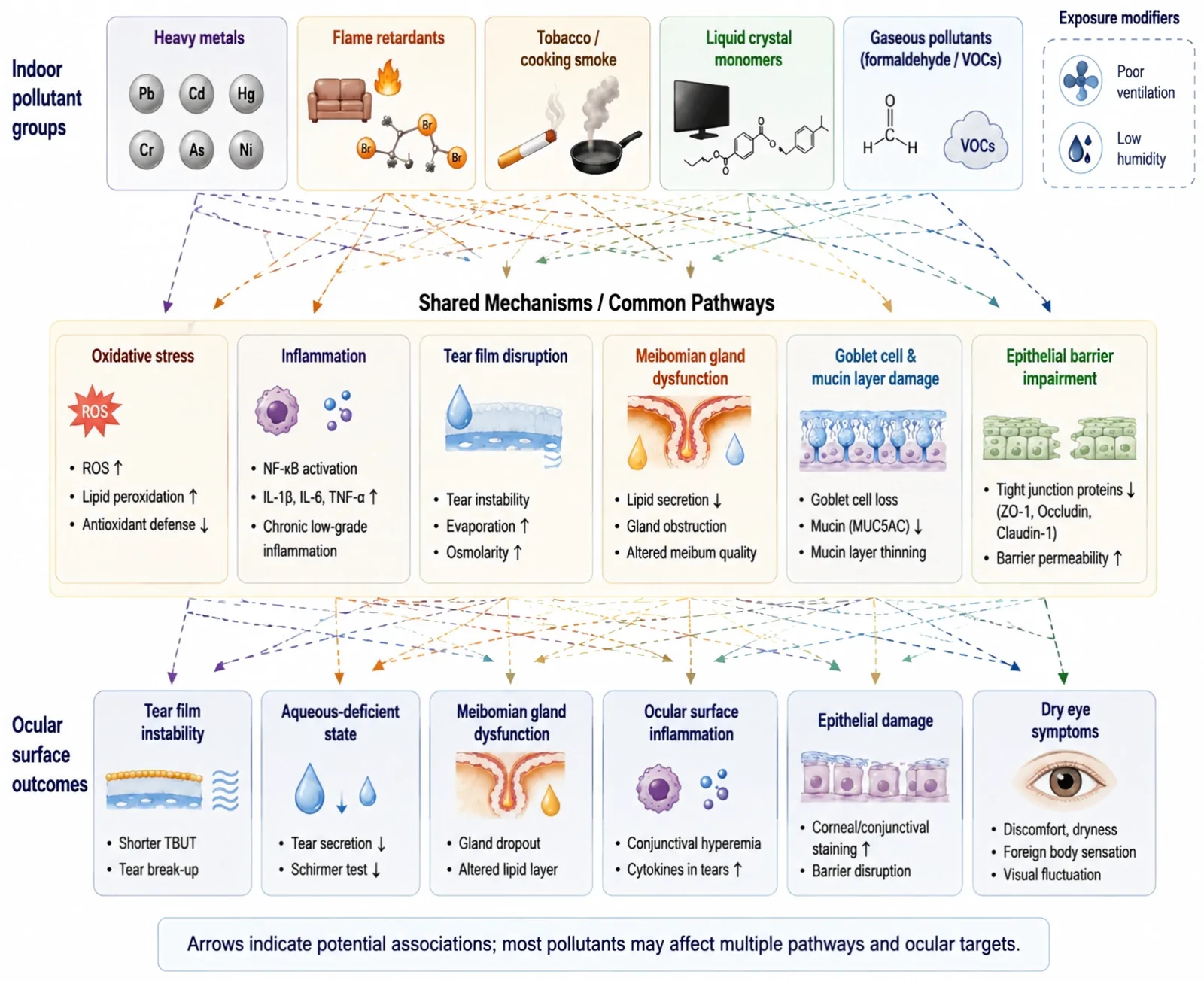
Potential associations between indoor environmental exposures and ocular surface structures in DED. The arrows indicate possible links suggested by current evidence. In practice, most pollutants may affect multiple overlapping pathways and ocular targets rather than acting through a single, exclusive mechanism. The figure is intended as a simplified schematic for illustrative purposes and does not represent a definitive causal map.

The findings were analyzed based on how directly each exposure matched the clinical criteria for diagnosing DED. Direct evidence was characterized as research documenting the diagnosis of DED, standardized symptoms of the condition, measurements such as tear break-up time (TBUT), results from the Schirmer test, assessments of ocular surface staining, evaluations of meibomian gland function, levels of inflammatory markers in tears, or other validated endpoints related to the ocular surface. Indirect indications encompassed biomarker correlations, research on similar autoimmune conditions affecting the lacrimal system, non-ocular toxicology data, indoor dust prevalence analyses, and biologically plausible mechanistic insights that have not yet been confirmed in populations with DED. This approach was adopted to avoid overinterpreting emerging pollutants with limited direct DED evidence. Figure 1 presents a simplified schematic of potential associations between indoor environmental exposures and ocular surface structures, with the understanding that most pollutants may affect multiple pathways and targets. 

## 2. Indoor Particulate Matter, Gaseous Pollutants, and Volatile Organic Compounds

### 2.1. Indoor Exposure Characteristics

The quality of indoor air is affected by internal sources, as well as the intrusion of pollutants from outside. Fine particles (such as PM2.5 and PM10), nitrogen dioxide, reaction byproducts from ozone, carbon monoxide, formaldehyde, benzene, acetaldehyde, terpenes, and emissions from cleaning agents, along with various other volatile organic compounds (VOCs), can accumulate indoors when emission levels are elevated or when there is insufficient ventilation [7,9]. The levels of indoor pollutants can differ significantly depending on factors such as the age of the building, the type of furniture and decoration materials used, cleaning and disinfection routines, cooking habits, the number of occupants, ventilation rates, temperature, and humidity levels [10].

### 2.2. Ocular Surface Mechanisms

Particulate matter and reactive gaseous contaminants can interact directly with the eye’s surface, disrupting the stability of tear film balance [11]. Evidence from experimental and epidemiological studies indicates that air pollution may contribute to oxidative stress [12], trigger inflammatory pathways, elevate tear osmolarity, and disrupt the barrier function of the corneal and conjunctival epithelium [10,13]. These outcomes align with the well-documented vicious cycle of DED, where the instability of the tear film and increased osmolarity exacerbate epithelial stress, trigger the release of cytokines, activate matrix metalloproteinases (MMPs), and promote the recruitment of inflammatory cells. PM2.5 exposure has been shown to significantly affect tear film parameters, including tear break-up time and Schirmer test values [14].

### 2.3. Clinical Relevance

Research on offices and buildings has associated suboptimal indoor air conditions, high levels of volatile organic compounds (VOCs), insufficient humidity, and poor airflow with eye irritation and symptoms [15]. Reduced humidity may promote the evaporation of tears and disrupt the stability of the lipid layer, while overly ventilated environments with dry air can potentially worsen symptoms if moisture levels are not adequately maintained [6,16]. The most reasonable clinical interpretation suggests that indoor air pollutants and specific microenvironmental factors could worsen ocular symptoms and tear stability, especially in individuals with pre-existing tear film instability, MGD, contact lens wear, or extended exposure to digital screens.

## 3. The Relationship Between Indoor Smoke Pollution and DED

### 3.1. Composition and Exposure Characteristics of Indoor Smoke

Indoor smoke pollution is caused by factors such as smoking, secondhand smoke [17], thirdhand smoke (tobacco residues that persist on indoor surfaces and in dust after active smoking has ceased) [18], emissions from cooking, burning incense, and the use of solid fuels for combustion. A meta-analysis has confirmed that smoking is significantly associated with an increased risk of DED [19]. Cigarette smoke contains fine particles, nicotine, aldehydes, polycyclic aromatic hydrocarbons, reactive oxidants, and metals. High-temperature cooking releases particulate matter, aldehydes, and various oxidative substances [7]. These hazardous compounds can reach the eyes either directly on the ocular surface or through the bloodstream after being inhaled. This makes indoor smoke an important environmental risk factor for DED and other ocular surface disorders.

### 3.2. Effects of Smoke on Tear Film Lipid Layer and Tear Secretion

Free radicals and oxidants from tobacco combustion damage the structure and function of the tear film lipid layer, making it thinner and changing its lipid makeup [20]. Clinical studies show that lipid layer thickness and tear break-up time (TBUT) decrease soon after smoking, which means that smoke rapidly harms tear film stability. Long-term smokers have persistently thinner lipid layers and worse DED, suggesting a cumulative effect. Smoking is also closely linked to meibomian gland atrophy and dysfunction [20]. Beyond smoking, dyslipidemia has been identified as a significant risk factor for MGD, with a systematic review and meta-analysis showing that high total cholesterol and high triglycerides are strongly associated with MGD prevalence [3]. The smoking index correlates negatively with gland number, size, and secretion [20]. Also, toxic substances in tobacco smoke can injure lacrimal gland tissue and lower tear production. This is supported by findings showing that smokers have much lower Schirmer test values than non-smokers [19].

### 3.3. Eye Surface Inflammation and Epithelial Damage Induced by Smoke

Smoke exposure triggers immune responses on the ocular surface and leads to long-lasting inflammation. Chronic smokers have significantly higher levels of matrix metalloproteinase 9 (MMP-9) in their tears, as demonstrated in a recent clinical study of 35 chronic smokers and 50 non-smokers [20,21]. Smoke also activates the nuclear factor kappa-B (NF-κB) pathway in human corneal epithelial cells, which promotes the release of pro-inflammatory cytokines (IL-1β and IL-6) and worsens inflammation [22]. The oxidative stress caused by smoke exceeds what the ocular surface antioxidant system can handle, resulting in cell death, as recently discovered, and ferroptosis in corneal epithelial cells, a process marked by lipid peroxidation and iron buildup [22]. Long-term smoke exposure also reduces goblet cell density, disrupting the mucus layer and further destabilizing the tear film.

### 3.4. Epidemiological Evidence and Clinical Studies

Symptoms such as dryness, foreign body sensation, burning, and visual fluctuation are positively linked to the amount of smoke exposure, consistent with findings from a systematic review and meta-analysis [19]. These symptom-based studies complement evidence from clinical studies that used objective DED diagnostic criteria. Acute smoking can cause a rapid decrease in the thickness of the lipid layer and the tear film rupture time, while chronic smoking is associated with the continuous deterioration of tear film stability and the progressive aggravation of DED.

### 3.5. Prevention and Intervention Recommendations

When documenting the environmental history of patients with chronic DED, factors such as smoking habits, exposure to second-hand smoke, and exposure to cooking fumes must be considered. The relevant measures should mainly include advocating smoking cessation, formulating policies to ensure a smoke-free indoor environment, improving kitchen ventilation systems, reducing exposure to high-temperature frying, and avoiding contact with combustion sources in poorly ventilated areas. These measures have extremely low risks and are in line with public health guidelines.

## 4. The Relationship Between Indoor Heavy Metal Pollution and DED

### 4.1. Sources and Indoor Exposure Characteristics of Heavy Metals

Toxic elements like lead, cadmium, copper, zinc, arsenic, chromium, nickel, and mercury can accumulate in indoor dust due to factors such as the intrusion of outdoor pollutants, industrial processes, vehicle emissions, coal burning, construction materials, electronic equipment, and domestic pollution sources [7]. Humans can be exposed through inhalation of airborne particles, swallowing of accumulated dust, or skin contact. Professionals including welders, employees involved in battery production, workers in metallurgy, and individuals engaged in electronic waste recycling are likely to encounter greater exposure levels than the general public [23,24].

### 4.2. Mechanisms of Heavy Metal-Induced Damage to the Tear Film Lipid Layer

Direct epidemiological data linking heavy metal exposure with clinically diagnosed DED are sparse but suggestive of an association [7]. Studies have reported associations between trace element levels in blood, urine, or tears and DED metrics such as TBUT, ocular symptoms, tear inflammatory markers, and work-related ocular surface damage. However, many studies are cross-sectional and struggle to separate heavy metal effects from co-occurring dust, fumes, solvents, heat exposure, smoking, and workplace conditions [24]. Mechanistically, toxic metals can induce oxidative stress, mitochondrial and endoplasmic reticulum dysfunction, lipid peroxidation, DNA damage, and inflammatory signaling [25,26]. Therefore, heavy metals should be regarded as a potential contributing factor to ocular surface damage, rather than an independent cause of definite DED.

### 4.3. Heavy Metal-Induced Conjunctival and Corneal Epithelial Injury

The cornea and conjunctiva make up the eye’s first line of defense. If this barrier breaks down, the eye becomes vulnerable. Heavy metals can damage this barrier in several ways. For instance, nickel at environmentally relevant concentrations has been shown to induce oxidative damage and apoptosis in human corneal epithelial cells [25]. Heavy metals also cause oxidative stress in epithelial cells, which leads to mitochondrial and endoplasmic reticulum dysfunction. Too many reactive oxygen species (ROS) are produced, causing cells to die. When cells are exposed to a mix of heavy metals, ROS levels increase, enzymes like superoxide dismutase (SOD) and catalase (CAT) become less active, and lipids and DNA are damaged. Heavy metals also bring more inflammatory cells (neutrophils and macrophages) into the tissue and raise the levels of inflammatory cytokines like IL-1β, IL-6, and TNF-α [25,26]. This makes the epithelial barrier even weaker. Heavy metals also cause reductions in the levels of tight junction proteins such as ZO-1, so the eye becomes more easily infected or irritated [7,26]. In patients, heavy metal damage to the eye surface causes irritation, burning, a feeling of something in the eye, and blurry vision. Severe or prolonged exposure can cause corneal epithelial breakdown, stromal haze, and permanent visual loss.

### 4.4. Epidemiological Evidence and Clinical Relevance

In settings with high exposure, heavy metals may cause eye discomfort, burning, foreign body sensation, vision changes, excessive tearing, or dryness. A study of shipyard welders found that tear lead levels were significantly associated with higher OSDI and SPEED scores, as well as reduced meibomian gland area [23]. When treating patients with ongoing ocular surface symptoms, doctors should take an occupational history, especially if the symptoms become worse at work or are linked to noticeable dust or fume exposure [23,24]. Preventive steps include controlling the source of contamination, using local exhaust ventilation, wearing personal protective equipment, monitoring the environment, and carrying out standard DED diagnostic tests, particularly for workers with known metal exposure [24]. These tests include symptom questionnaires, TBUT, ocular surface staining, Schirmer testing, and meibomian gland assessment.

## 5. Impact of Flame Retardant Contamination on Ocular Surface Health

### 5.1. Indoor Distribution and Exposure Pathways of Flame Retardants

Fire-resistant chemicals are added to upholstery foam, fabrics, electronic devices, polymers, and construction materials. Restrictions and phase-outs of polybrominated diphenyl ethers have been implemented in numerous areas, whereas organophosphate esters (OPEs), such as triphenyl phosphate and tris(1,3-dichloro-2-propyl) phosphate, are commonly found in indoor environments, including air and dust [27]. Human exposure mainly occurs through the intake of dust, inhalation, skin contact, and behavioral factors like hand-to-mouth behavior [27]. Certain occupational groups and children may face elevated levels of exposure.

### 5.2. Flame Retardant-Induced Tear Film Dysfunction

There is limited direct clinical evidence connecting exposure to flame retardants with DED. Flame retardants might influence ocular surface stability via toxic and immune-related mechanisms, yet their direct role in contributing to DED has not been confirmed. A case–control study has identified links between serum OPE levels and Sjögren syndrome, a condition marked by impaired lacrimal and salivary gland function [28]. Nonetheless, this observation should not be interpreted as conclusive proof of causation of DED. The immunological basis for keratoconjunctivitis sicca in systemic autoimmune diseases has been extensively reviewed, highlighting how innate immune sensors and systemic immune dysregulation can predispose to dry eye disease [29]. However, this cross-sectional observation cannot establish causality, and residual confounding by co-occurring exposures (e.g., other flame retardants, plasticizers, or indoor dust constituents) cannot be excluded.

### 5.3. Toxic Effects of Flame Retardants on Ocular Surface Cells

Toxicology research indicates that certain flame retardants have the potential to trigger oxidative stress, disrupt mitochondrial function, promote cell apoptosis, initiate inflammatory responses, and influence extracellular matrix alterations [30]. These processes intersect with established pathways of dry eye disease, such as epithelial strain, dysfunction of goblet cells, inflammation in the lacrimal glands, and instability of the tear film. Future research should investigate the relationship between indoor dust OPE concentrations or urinary OPE metabolites and standardized indicators and symptoms of DED, taking into account factors such as age, gender, smoking habits, contact lens usage, humidity, screen exposure, and the presence of co-pollutants.

### 5.4. Association Between Flame Retardant Exposure and Clinical Manifestations of DED

While direct clinical evidence specific to DED is still scarce, exposure to organophosphate flame retardants and associated indoor dust pollutants could hold clinical significance in occupational environments or spaces with inadequate ventilation. Existing evidence suggests careful consideration: exposure to flame retardants may be a potential factor in ocular surface irritation and tear film instability, rather than a definitive standalone cause of DED.

## 6. Emerging Pollutants: Impact of Liquid Crystal Monomers on DED

### 6.1. Sources and Exposure Pathways of Liquid Crystal Monomers

Liquid crystal monomers (LCMs) are synthetic compounds with excellent optoelectronic properties, widely used in LCDs and other electronic display devices, including computer monitors, smartphones, and televisions. With widespread use and frequent replacement of these electronic products, LCMs are gradually released into indoor environments, emerging as a new class of environmental pollutants [31,32]. This suggests that electronics and e-waste handling may contribute to indoor contamination [33]. Exposure to certain fluorinated LCMs can occur via dust ingestion, dermal contact, inhalation of residue-carrying particles, and possibly breastfeeding in infants [34].

### 6.2. Toxic Effects of Liquid Crystal Monomers on the Tear Film and Ocular Surface Cells

It would be premature to characterize LCMs as definitive DED-inducing VOCs or to assert that they directly compromise the tear film lipid layer in humans. Recent LCM studies have primarily focused on environmental occurrence, exposure assessment, bioaccumulation, and concerns related to developmental or systemic toxicity [33]. Direct investigations of LCMs in ocular surface cells, the tear film lipid layer, meibomian glands, or clinically diagnosed DED remain lacking at present.

### 6.3. Research Hypotheses and Future Directions

It is scientifically reasonable to suspect that some liquid crystal monomers (LCMs) could be toxic to the ocular surface, but this idea has not been proven. Future studies should focus on testing this possibility. Useful approaches would include experiments using human corneal or conjunctival epithelial cells, exposure assessments that link LCM levels in indoor dust to clinical signs of DED, and mechanistic studies that look for the oxidative or inflammatory pathways involved.

## 7. Impact of Indoor Air Contaminants (e.g., Formaldehyde and CO_2_) on Ocular Health

### 7.1. Formaldehyde: Sources, Exposure, and Ocular Irritation Mechanisms

Indoor gaseous pollutants, especially volatile organic compounds (VOCs) like formaldehyde, can irritate and damage the ocular surface epithelium. Formaldehyde is common in building materials, furniture, and disinfectants. Levels are often high in healthcare settings, newly renovated homes, and enclosed spaces. Studies show that people exposed to high formaldehyde levels often report eye irritation, burning, and dryness. Formaldehyde directly stimulates epithelial cells on the eye surface, triggering inflammation and cell damage [35]. This leads to poorer tear film stability and more severe DED.

### 7.2. The Ocular Surface Effects of CO_2_

Indoor carbon dioxide (CO_2_) is not a direct eye irritant, but it is a useful indicator of how well a room is ventilated. When ventilation is poor, CO_2_ levels increase, and other pollutants generated by people inside also accumulate. Studies have linked elevated indoor CO_2_ levels to eye discomfort. In one study of 417 office workers, every 100 ppm increase in the indoor–outdoor CO_2_ difference (dCO_2_) raised the odds of dry eyes, tiredness, and dizziness by 10–16% [36]. Another study reported a similar dose–response relationship: each 100 ppm rise in dCO_2_ increased the risk of dry eyes and sore throat by 8–21% [37]. When CO_2_ levels exceeded 800 ppm, workers were more likely to report ocular irritation symptoms (OR = 1.8, 95% CI: 1.2–2.8) [38]. Also, wearing a mask for a long time may affect the ocular surface through altered local airflow and increased tear evaporation, as discussed in Section 8.1 [39]. Carbon dioxide (CO_2_) levels serve as a useful indicator of ventilation efficacy, and elevated CO_2_ concentrations are associated with symptoms of ocular irritation. However, such symptom-based findings reflect ocular discomfort rather than clinically diagnosed DED. CO_2_ itself is not a pollutant but rather an indicator that other pollutants may accumulate under poor ventilation conditions [40].

### 7.3. Synergistic Effects with Other Pollutants

VOCs and their breakdown products in indoor air not only directly irritate the eye surface but may also make eye disease worse by forming secondary organic aerosols. Interactions between temperature, humidity, and gas pollutants also affect eye health. Lower humidity or temperature is linked to worse dry eye symptoms, while pollutants like NO_2_, O_3_, and PM2.5 are clearly tied to eye inflammation markers [16,41]. This means that management of indoor air quality requires consideration of how multiple gas pollutants and environmental factors work together to affect eye health.

### 7.4. Clinical Significance and Prevention

In short, VOCs such as formaldehyde directly irritate the eye surface, causing inflammation and damage, which leads to discomfort and dry eye symptoms. High CO_2_ levels mostly indicate that ventilation is poor, which then impacts tear film stability and eye comfort. To reduce ocular discomfort and prevent DED, it is essential to control indoor air quality by lowering formaldehyde levels and improving ventilation. The World Health Organization (WHO) has established an indoor air quality guideline for formaldehyde of 0.1 mg/m^3^ (0.08 ppm) for all 30 min periods at lifelong exposure to protect against eye and nasal irritation and long-term health effects [42,43].

## 8. Physical Microenvironmental Factors: Mask Airflow, Humidity, and Ventilation

### 8.1. The Effect of Mask-Related Airflow on Tear Film Stability

Unlike the chemical contaminants discussed above, physical factors (mask airflow, humidity, and ventilation) affect the eye surface mainly through biomechanical and evaporative effects, not direct toxicity. Wearing a face mask can redirect exhaled air toward the eyes, changing local humidity, temperature, and tear evaporation rate. A comprehensive review has confirmed that face mask use is associated with increased dry eye disease symptoms, decreased tear break-up time, and corneal epithelial trauma and secretion of inflammatory markers [39]. Studies and reviews have found a link between dry eye symptoms or eye irritation and mask use, especially when masks do not fit well or when people already have DED or meibomian gland dysfunction, spend a long time using screens, or work in low-humidity settings [39]. This is mostly due to physical factors and the local environment: more airflow speeds up evaporation and disrupts tear film stability.

### 8.2. Low Humidity and Stress Evaporation

Low humidity increases evaporation stress, disrupts the lipid layer, and shortens tear film break-up time. In office and building studies, indoor humidity below 40% is consistently linked to more eye discomfort and worse DED symptoms [6,16]. A population-based study reported an association between residence in areas with relative humidity below 70% and higher OSDI scores, suggesting a possible link between moderate reductions in ambient humidity and dry eye symptoms, although the association was based on symptom classification rather than clinical DED diagnosis and requires further confirmation [44]. Temperature also affects tear film stability: high temperatures speed up tear evaporation, while low temperatures may increase the thickness of meibomian gland secretions, making it harder for the lipid layer to spread evenly. But indoor temperature changes are usually smaller than humidity changes, so there is not yet enough evidence to directly link indoor temperature to DED. Age-related changes in meibomian gland function, including gland atrophy and dropout, represent a major contributor to evaporative dry eye disease in older populations [4].

### 8.3. The Amplifying Effect of Insufficient Ventilation on Pollutant Accumulation

Poor ventilation allows higher concentrations of VOCs, airborne particles, and occupant-generated bioaerosols to accumulate indoors, thereby increasing the total exposure burden on the ocular surface [6,7].

### 8.4. Comprehensive Management Strategy

Therefore, preventing DED requires a broad approach that addresses both pollution sources and environmental conditions, rather than focusing on any single factor. Practical steps that complement source control include keeping indoor humidity between 40 and 60%, ensuring good ventilation without making the air too dry, and wearing well-fitted masks.

## 9. Integrated Mechanisms from Indoor Exposure to DED

### 9.1. Molecular Mechanism

Notwithstanding the considerable diversity in chemical composition, origin, and routes of exposure among distinct pollutant categories, they tend to exert their deleterious effects on the ocular surface through a limited repertoire of shared biological pathways. These encompass the generation of reactive oxygen species (ROS), lipid peroxidation, NF-κ B-mediated inflammatory signaling, secretion of cytokines, activation of matrix metalloproteinases (MMPs), disruption of epithelial tight junctions, programmed cell death, immune dysregulation, dysfunction of the meibomian or lacrimal glands, and deficiency of the mucin layer [45,46]. Emerging therapeutic strategies targeting oxidative stress, including nanomedicine-based approaches, are being actively explored for dry eye disease [47].

### 9.2. Biophysical and Mechanical Disruption of the Tear Film

Tears are to the ocular surface what blood is to internal organs—they nourish, protect, and maintain homeostasis [48]. For the tear film to function properly, it needs more than the right chemical composition; its physical properties are equally critical. The lipid layer must spread evenly and stay intact to slow evaporation [49,50,51]. The mucoaqueous gel needs to maintain viscosity and shear-thinning behavior so that it can coat the eye smoothly during blinks and then recover quickly to form a stable protective layer [52,53]. Indoor pollutants can disrupt these properties [54]. Oxidative damage from tobacco smoke or particulate matter can thin the lipid layer and accelerate evaporation [55]. Volatile organic compounds and other reactive agents may also affect the mucin network, although direct evidence for this pathway is still limited. Some experimental data suggest that environmental stressors, including low humidity and temperature changes, can alter mucin-related interfacial properties in model systems [52,56,57]. Whether these findings translate directly to VOC-induced mucin damage in humans remains a hypothesis requiring further investigation. The clinical result is often the same: tear film break-up occurs too soon—often in less than six seconds—well before the next blink [41,58,59,60]. This premature rupture leaves the corneal epithelium exposed to environmental insults, which may in turn activate the inflammatory and oxidative cascades described above. In this sense, physical breakdown of the tear film may be one of the earliest events in pollutant-induced DED, triggering rather than merely accompanying the later biochemical damage.

### 9.3. Pathological Outcome

**Figure 2:**
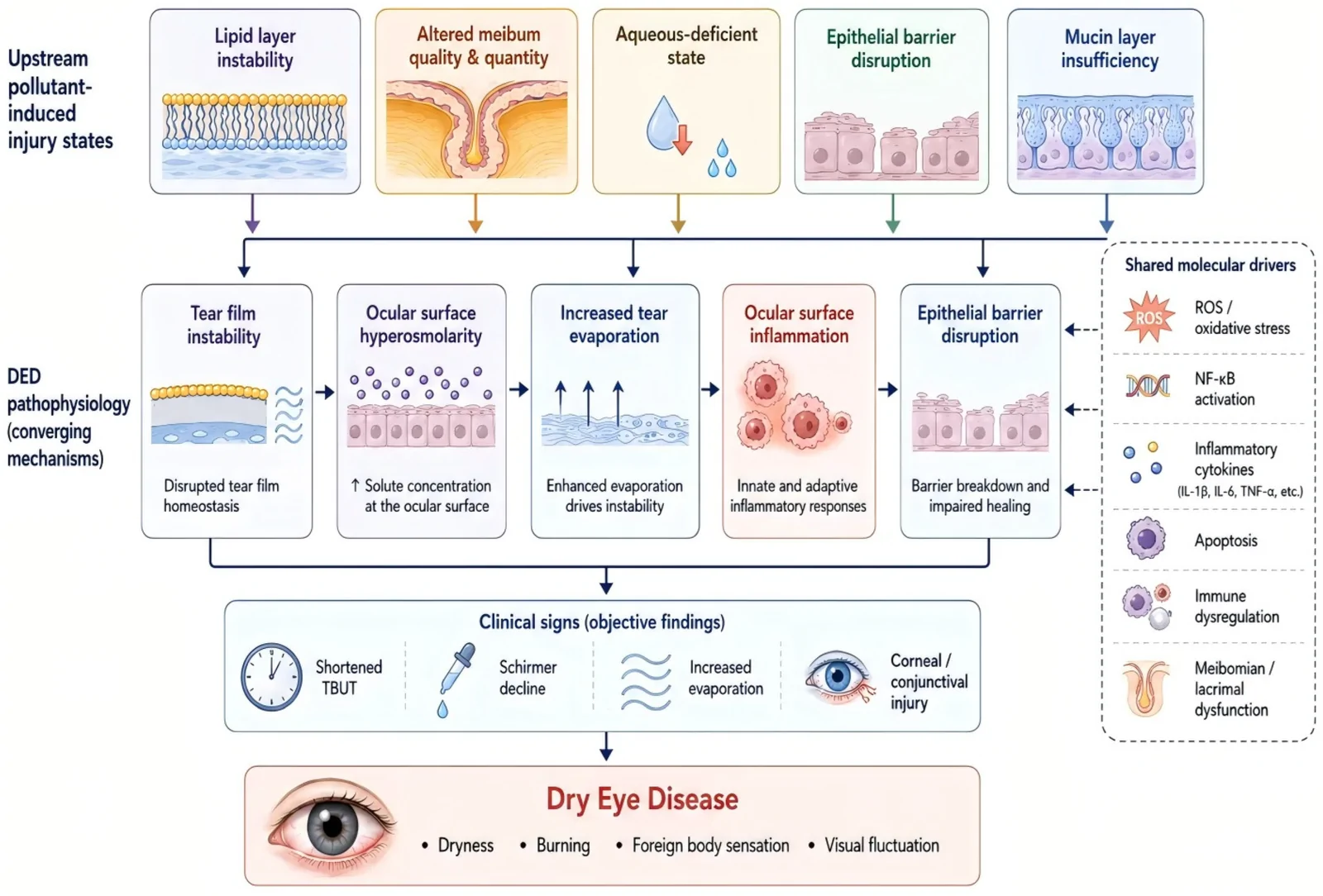
Mechanisms resulting in the development of DED. Injury caused by pollutants leads to tear film imbalance, increased osmolarity, excessive evaporation, inflammatory responses, and damage to the epithelial barrier, resulting in clinical manifestations and discomfort associated with DED.

What happens at the tissue level because of these molecular events? ROS cause lipid peroxidation, which breaks down the tear film lipid layer. NF-κB drives inflammation, which impairs meibomian and lacrimal gland function. These shared pathways explain why different indoor exposures can all lead to DED. When the lipid layer is damaged and meibum composition changes, tears evaporate faster. A reduction in tear production results in a tear volume insufficient to effectively dilute and clear ocular surface irritants. When the epithelial barrier is broken, inflammation becomes worse. When goblet cells are lost, mucin runs low, and the tear film cannot spread evenly. This cycle—tear film disruption, high osmolarity, inflammation, and epithelial damage—keeps going and explains why different indoor factors cause similar eye symptoms. Figure 2 shows how pollutant injury leads to tear film imbalance, high osmolarity, excessive evaporation, inflammation, and epithelial damage.

## 10. Clinical Impact and Prevention Strategies

### 10.1. Clinical Assessment

The evaluation of DED should routinely include an assessment of environmental factors, especially for individuals experiencing ongoing symptoms, work-related exposure, recurring flare-ups in certain environments, or limited improvement with conventional treatments [41,61]. Key areas encompass exposure to secondhand smoke and smoking directly, fumes from cooking, recent home renovations, newly purchased furniture, use of cleaning agents or disinfectants, workplace exposure to dust or fumes, high density of electronic devices, prolonged use of screens, wearing contact lenses, and airflow related to mask usage, as well as factors like ventilation, temperature, and humidity [15]. Clinical evaluation should follow standardized protocols and could involve the use of symptom surveys, tear break-up time (TBUT) tests, the Schirmer test, ocular surface staining, tear osmolarity measurements if accessible, MMP-9 analysis, and assessments of the meibomian glands. Brief environmental exposure screening questions for clinical practice may include those listed below.

These questions in Table 1 are a practical reference for clinical environmental history-taking and have not been validated as a diagnostic tool.

The thresholds provided in these questions are derived from previous environmental exposure studies and expert consensus, and they should be interpreted as general guidance rather than strict clinical cutoffs. According to ANSI/ASHRAE Standard 55-2020, the upper humidity limit for thermal comfort is set at a humidity ratio of 0.012 (approximately 60–65% relative humidity, depending on temperature), with no specified lower limit for thermal comfort purposes [62]. Although field studies have identified 40–60% relative humidity as an optimal range for indoor environments [63], this threshold is more appropriately viewed as a practical guideline derived from general industry and public health recommendations, rather than a strict requirement imposed by the standard itself [62].

### 10.2. Prevention Strategies

Preventive efforts should prioritize source control, minimization of exposure, and improvement of the indoor environment. Effective strategies encompass the implementation of smoke-free indoor policies, promotion of smoking cessation, assurance of proper kitchen exhaust, reduction in emissions from high-temperature cooking, prudent use of cleaning products, enhancement of air circulation, control of formaldehyde and volatile organic compound (VOC) emission sources, maintenance of comfortable indoor humidity, and mitigation of visible dust accumulation. In occupational contexts, engineering controls, local exhaust ventilation, appropriate personal protective equipment, exposure assessments, and symptom monitoring are of paramount importance for workers handling metal particulates, welding emissions, flame retardants, or electronic waste [23]. A prospective study evaluating home environmental interventions in United States veterans demonstrated that modifying the indoor environment can improve DED symptoms and signs, supporting the clinical relevance of environmental management [64].

### 10.3. Therapeutic Implications

Environmental adjustments should complement, rather than supplant, standard DED treatments and may augment symptom management when integrated with conventional approaches. Based on DED phenotype and severity, management options may include artificial tears, lipid-containing lubricants, eyelid hygiene, warm compresses, anti-inflammatory agents, punctal occlusion, meibomian gland dysfunction (MGD) therapy, and management of systemic or autoimmune factors [65]. Individuals with environmentally triggered symptoms may derive benefit from tailored advice addressing pollutant sources, airflow patterns, blink habits during screen use, and optimal humidity levels.

## 11. Evidence Strength, Limitations, and Future Research Directions

### 11.1. Evidence Strength and Research Priorities

Not all pollutants have the same amount of evidence behind them. Table 2 provides a brief summary of the strength of evidence for each exposure and the recommended wording when discussing it. This is important because we do not want to overstate causal links, especially for emerging pollutants that have not been studied much in DED. The evidence grading shown in Table 2 reflects the strength of direct clinical evidence linking each exposure to DED and is not a measure of general toxicity. We classified studies as providing direct evidence if they assessed clinically diagnosed DED or used validated endpoints—standardized symptom scores, TBUT, Schirmer test results, ocular surface staining, meibomian gland function, or tear inflammatory markers. Studies reporting biomarker correlations, autoimmune conditions affecting the lacrimal system, non-ocular toxicology, indoor dust levels, or biologically plausible mechanisms without DED-specific outcomes were considered to provide indirect evidence. Where direct clinical data were limited, we avoided strong causal interpretations. This approach is intended to help readers distinguish well-supported associations from hypotheses that require further investigation.

### 11.2. Limitations of Current Evidence

Several limitations warrant candid acknowledgment. First, many studies are cross-sectional, so we cannot easily say what causes what. Second, different studies define DED in different ways. Some studies rely on self-reported symptoms (e.g., dryness and irritation) rather than validated diagnostic criteria such as the TFOS DEWS guidelines. Findings of symptom-based studies should therefore be distinguished from those of studies that used clinical DED diagnoses, as the former reflect ocular discomfort rather than confirmed disease. This distinction is important for interpreting the evidence and for avoiding overestimation of associations between environmental exposures and DED. Third, indoor air contains a mix of many substances. Experiments examining one pollutant at a time do not reflect real-world conditions. Fourth, few studies have combined personal exposure data, indoor air quality measurements, eye surface biomarkers, and full clinical assessments. Finally, people vary. Factors such as age, sex, hormones, autoimmune diseases, contact lens use, job exposures, blink rate, screen time, and health status of the meibomian glands can change how indoor environments affect an individual’s eyes [67].

### 11.3. Future Research Directions

Several important avenues remain for future investigation. Researchers should conduct prospective cohort studies and use standard DED criteria, track each person’s exposures, and measure indoor factors repeatedly over time. Approaches that can account for coexposure to multiple pollutants—for example, mixture analysis methods such as weighted quantile sum regression or Bayesian kernel machine regression—may help to better characterize the combined effects of interrelated pollutants. Mechanistic studies are required to understand how specific indoor factors affect the meibomian glands, goblet cells, tight junctions, tear cytokines, lipid profiles, and nerve pathways. Intervention studies are also required to determine whether measures like better ventilation, the use of air purifiers, humidity control, smoke elimination, and source reduction can help DED patients in a meaningful way.

## 12. Conclusions and Future Perspectives

Exposure to indoor environments is a significant and adjustable factor in the prevention and treatment of DED. Available evidence most convincingly links outcomes related to DED with exposure to tobacco smoke, fine particles in the air, poor indoor air conditioning, insufficient humidity, and suboptimal ventilation. Toxic substances like heavy metals, flame retardants, and LCMs may contribute to damage of the ocular surface, though direct clinical data are scarce and must be interpreted with care. Pollutant categories share common mechanisms such as oxidative stress, inflammation activation, disruption of the lipid layer, dysfunction of the meibomian and lacrimal glands, impairment of the mucin layer, and damage to the epithelial barrier. Combining environmental history analysis, comprehensive ocular surface evaluations, and strategies to minimize indoor exposure could enhance personalized treatment for DED. This approach also lays a solid foundation for future clinical and public health measures centered on exposure management. Therefore, clinicians are encouraged to routinely inquire about indoor environmental exposures (smoking, cooking fumes, recent renovation, mask use, ventilation, and humidity) when managing refractory DED. For example, randomized controlled trials could evaluate the effect of portable air purifiers with HEPA and activated carbon filters on DED symptoms and signs in office workers, or test whether humidification to maintain 50% relative humidity improves tear film stability in low-humidity environments.

## Figures and Tables

**Table 1:** Suggested environmental exposure screening questions for clinical practice.

Number	Question
1	Do you smoke, or are you regularly exposed to secondhand smoke?
2	Do you cook with high-temperature methods (e.g., frying, wok cooking) without adequate ventilation?
3	Has your home or workplace been renovated recently?
4	Do you wear a face mask for prolonged periods?
5	Do you work with metals, solvents, or electronic waste?
6	Is your indoor relative humidity often below 40% or above 60%?

**Table 2:** Strength of evidence linking indoor environmental pollutants to DED.

Exposure Category	Main DED-Related Evidence	Recommended Wording	Key References
Tobacco smoke and indoor smoke	Clinical, epidemiological, and mechanistic evidence	Associated with DED/MGD and may aggravate symptoms	[17,19,20]
Particulate matter and ambient/indoor pollution	Epidemiological and experimental evidence	Associated with DED risk and ocular surface inflammation	[10,11,12,13]
Low humidity, poor ventilation, VOCs, and formaldehyde	Office/building studies and irritant toxicology	May worsen ocular discomfort and tear film instability	[6,15,16]
Mask-associated airflow and indoor microenvironment	Clinical reports and systematic reviews	May aggravate evaporation-related symptoms in susceptible individuals	[39]
Heavy metals	Biomarker/occupational studies and toxicology	Possible contributing factor; data remain limited and inconclusive	[23,25,26]
Flame retardants	Studies on indoor dust exposure and toxicological impacts; minimal evidence tied to specific diseases	Biologically plausible but not established as an independent DED cause	[27,28,30]
Liquid crystal monomers	Emerging exposure data; direct clinical evidence is currently inadequate	Current evidence is insufficient to establish a causal relationship	[31,33,66]

## Data Availability

No new data were created or analyzed in this study. Data sharing is not applicable to this article.

## References

[1] Wolffsohn J.S., Benítez-Del-Castillo J.M., Loya-Garcia D., Inomata T., Iyer G., Liang L., Pult H., Sabater A.L., Starr C.E., Vehof J. (2025). TFOS DEWS III: Diagnostic Methodology. Am. J. Ophthalmol..

[2] Stapleton F., Argüeso P., Asbell P., Azar D., Bosworth C., Chen W., Ciolino J.B., Craig J.P., Gallar J., Galor A. (2025). TFOS DEWS III: Digest. Am. J. Ophthalmol..

[3] Tomioka Y., Kitazawa K., Yamashita Y., Numa K., Inomata T., Hughes J.B., Soda R., Nakamura M., Suzuki T., Yokoi N. (2023). Dyslipidemia Exacerbates Meibomian Gland Dysfunction: A Systematic Review and Meta-Analysis. J. Clin. Med..

[4] Moreno I., Verma S., Gesteira T.F., Coulson-Thomas V.J. (2023). Recent advances in age-related meibomian gland dysfunction (ARMGD). Ocul. Surf..

[5] Patel S., Mittal R., Kumar N., Galor A. (2023). The environment and dry eye-manifestations, mechanisms, and more. Front. Toxicol..

[6] Alves M., Asbell P., Dogru M., Giannaccare G., Grau A., Gregory D., Kim D.H., Marini M.C., Ngo W., Nowinska A. (2023). TFOS Lifestyle Report: Impact of environmental conditions on the ocular surface. Ocul. Surf..

[7] Yang D.L., Zhang Z.N., Liu H., Yang Z.Y., Liu M.M., Zheng Q.X., Chen W., Xiang P. (2023). Indoor air pollution and human ocular diseases: Associated contaminants and underlying pathological mechanisms. Chemosphere.

[8] Perez V.L., Chen W., Craig J.P., Dogru M., Jones L., Stapleton F., Wolffsohn J.S., Sullivan D.A. (2026). TFOS DEWS III: Executive Summary. Am. J. Ophthalmol..

[9] Muruganandam N., Mahalingam S., Narayanan R., Rajadurai E. (2023). Meandered and muddled: A systematic review on the impact of air pollution on ocular health. Environ. Sci. Pollut. Res. Int..

[10] Han J.H., Amri C., Lee H., Hur J. (2024). Pathological Mechanisms of Particulate Matter-Mediated Ocular Disorders: A Review. Int. J. Mol. Sci..

[11] Upaphong P., Thonusin C., Wanichthanaolan O., Chattipakorn N., Chattipakorn S.C. (2024). Consequences of exposure to particulate matter on the ocular surface: Mechanistic insights from cellular mechanisms to epidemiological findings. Environ. Pollut..

[12] Yu D., Cai W., Shen T., Wu Y., Ren C., Li T., Hu C., Zhu M., Yu J. (2023). PM(2.5) exposure increases dry eye disease risks through corneal epithelial inflammation and mitochondrial dysfunctions. Cell Biol. Toxicol..

[13] Iqbal S., Ramini A., Kaja S. (2025). Impact of particulate matter and air pollution on ocular surface disease: A systematic review of preclinical and clinical evidence. Ocul. Surf..

[14] Kausar S., Tongchai P., Yadoung S., Sabir S., Pata S., Khamduang W., Chawansuntati K., Yodkeeree S., Wongta A., Hongsibsong S. (2024). Impact of fine particulate matter (PM(2.5)) on ocular health among people living in Chiang Mai, Thailand. Sci. Rep..

[15] Huang A., Janecki J., Galor A., Rock S., Menendez D., Hackam A.S., Jeng B.H., Kumar N. (2020). Association of the Indoor Environment With Dry Eye Metrics. JAMA Ophthalmol..

[16] Song M.S., Lee Y., Paik H.J., Kim D.H. (2023). A Comprehensive Analysis of the Influence of Temperature and Humidity on Dry Eye Disease. Korean J. Ophthalmol..

[17] Zhang Y., Zhang X.J., Yuan N., Wang Y.M., Ip P., Chen L.J., Tham C.C., Pang C.P., Yam J.C. (2023). Secondhand smoke exposure and ocular health: A systematic review. Surv. Ophthalmol..

[18] Jacob P., Benowitz N.L., Destaillats H., Gundel L., Hang B., Martins-Green M., Matt G.E., Quintana P.J., Samet J.M., Schick S.F. (2017). Thirdhand Smoke: New Evidence, Challenges, and Future Directions. Chem. Res. Toxicol..

[19] Tariq M.A., Amin H., Ahmed B., Ali U., Mohiuddin A. (2022). Association of dry eye disease with smoking: A systematic review and meta-analysis. Indian J. Ophthalmol..

[20] Carreira A.R., Rodrigues-Barros S., Silva J.C., de Almeida M.F., Machado I., Cardoso J.N., Campos N. (2023). Tobacco effects on ocular surface, meibomian glands, and corneal epithelium and the benefits of treatment with a lipid-based lubricant. Graefes Arch. Clin. Exp. Ophthalmol..

[21] Güven S., Arıkan S., Uysal M.E., Cakır I. (2025). A non-invasive Schirmer strip-based analysis of tear cotinine and MMP-9 levels in chronic smokers. Cont. Lens Anterior Eye.

[22] Otsu W., Ishida K., Chinen N., Nakamura S., Shimazawa M., Tsusaki H., Hara H. (2021). Cigarette smoke extract and heated tobacco products promote ferritin cleavage and iron accumulation in human corneal epithelial cells. Sci. Rep..

[23] Chen Y.J., Chen Y.Y., Lai C.H. (2022). Clinical association between trace elements of tear and dry eye metrics. Sci. Rep..

[24] Liou Y.H., Chen Y.J., Chen W.L., Li K.Y., Chou T.Y., Huang Y.C., Wang C.C., Lai C.H. (2022). Associations between Biomarkers of Metal Exposure and Dry Eye Metrics in Shipyard Welders: A Cross-Sectional Study. Int. J. Environ. Res. Public Health.

[25] Zhang Z.N., Liu H., Liu M.M., Yang D.L., Bi J., Chen Q.Q., Chen W., Xiang P. (2022). Effects of Nickel at Environmentally Relevant Concentrations on Human Corneal Epithelial Cells: Oxidative Damage and Cellular Apoptosis. Biomolecules.

[26] Yang Z.Y., Liu H., Li J.Y., Bao Y.B., Yang J., Li L., Zhao Z.Y., Zheng Q.X., Xiang P. (2024). Road dust exposure and human corneal damage in a plateau high geological background provincial capital city: Spatial distribution, sources, bioaccessibility, and cytotoxicity of dust heavy metals. Sci. Total Environ..

[27] Peng Y., Shi C., Wang C., Li Y., Zeng L., Zhang J., Huang M., Zheng Y., Chen H., Chen C. (2023). Review on typical organophosphate diesters (di-OPEs) requiring priority attention: Formation, occurrence, toxicological, and epidemiological studies. J. Hazard. Mater..

[28] Liao K., Zhao Y., Qu J., Yu W., Hu S., Fang S., Zhao M., Jin H. (2023). Organophosphate esters concentrations in human serum and their associations with Sjögren syndrome. Environ. Pollut..

[29] Stern M.E., Theofilopoulos A.N., Steven P., Niederkorn J.Y., Fox R., Calonge M., Scheid C., Pflugfelder S.C. (2024). Immunologic basis for development of keratoconjunctivitis sicca in systemic autoimmune diseases: Role of innate immune sensors. Ocul. Surf..

[30] Zhang Z.N., Yang D.L., Liu H., Bi J., Bao Y.B., Ma J.Y., Zheng Q.X., Cui D.L., Chen W., Xiang P. (2023). Effects of TCPP and TCEP exposure on human corneal epithelial cells: Oxidative damage, cell cycle arrest, and pyroptosis. Chemosphere.

[31] Su H., Wang Y., Wu J., Gao P., Su G., Zhang H. (2024). A comparative study on contamination profiles of liquid crystal monomers (LCMs) between outdoor and indoor dusts, and the assessment of health risk of human exposure. Chemosphere.

[32] Wang Y., Jin Q., Lin H., Xu X., Leung K.M.Y., Kannan K., He Y. (2024). A review of liquid crystal monomers (LCMs) as emerging contaminants: Environmental occurrences, emissions, exposure routes and toxicity. J. Hazard. Mater..

[33] Stadelmann B., Leonards P.E.G., Brandsma S.H. (2024). A new class of contaminants of concern? A comprehensive review of liquid crystal monomers. Sci. Total Environ..

[34] Yang R., Wang X., Niu Y., Chen X., Shao B. (2023). Fluorinated liquid-crystal monomers in paired breast milk and indoor dust: A pilot prospective study. Environ. Int..

[35] Vazquez-Ferreiro P., Carrera Hueso F.J., Alvarez Lopez B., Diaz-Rey M., Martinez-Casal X., Ramón Barrios M.A. (2019). Evaluation of formaldehyde as an ocular irritant: A systematic review and Meta-analysis. Cutan. Ocul. Toxicol..

[36] Lu C.Y., Lin J.M., Chen Y.Y., Chen Y.C. (2015). Building-Related Symptoms among Office Employees Associated with Indoor Carbon Dioxide and Total Volatile Organic Compounds. Int. J. Environ. Res. Public Health.

[37] Erdmann C.A., Apte M.G. (2004). Mucous membrane and lower respiratory building related symptoms in relation to indoor carbon dioxide concentrations in the 100-building BASE dataset. Indoor Air.

[38] Tsai D.H., Lin J.S., Chan C.C. (2012). Office workers’ sick building syndrome and indoor carbon dioxide concentrations. J. Occup. Environ. Hyg..

[39] Burgos-Blasco B., Arriola-Villalobos P., Fernandez-Vigo J.I., Oribio-Quinto C., Ariño-Gutierrez M., Diaz-Valle D., Benitez-Del-Castillo J.M. (2023). Face mask use and effects on the ocular surface health: A comprehensive review. Ocul. Surf..

[40] Dimitroulopoulou S., Dudzińska M.R., Gunnarsen L., Hägerhed L., Maula H., Singh R., Toyinbo O., Haverinen-Shaughnessy U. (2023). Indoor air quality guidelines from across the world: An appraisal considering energy saving, health, productivity, and comfort. Environ. Int..

[41] Choi Y.H., Song M.S., Lee Y., Paik H.J., Song J.S., Choi Y.H., Kim D.H. (2024). Adverse effects of meteorological factors and air pollutants on dry eye disease: A hospital-based retrospective cohort study. Sci. Rep..

[42] Nielsen G.D., Larsen S.T., Wolkoff P. (2017). Re-evaluation of the WHO (2010) formaldehyde indoor air quality guideline for cancer risk assessment. Arch. Toxicol..

[43] Nielsen G.D., Larsen S.T., Wolkoff P. (2013). Recent trend in risk assessment of formaldehyde exposures from indoor air. Arch. Toxicol..

[44] Martin R. (2023). Symptoms of dry eye related to the relative humidity of living places. Cont. Lens Anterior Eye.

[45] Bu J., Liu Y., Zhang R., Lin S., Zhuang J., Sun L., Zhang L., He H., Zong R., Wu Y. (2024). Potential New Target for Dry Eye Disease-Oxidative Stress. Antioxidants.

[46] Bhujbal S., Rupenthal I.D., Steven P., Agarwal P. (2024). Inflammation in Dry Eye Disease-Pathogenesis, Preclinical Animal Models, and Treatments. J. Ocul. Pharmacol. Ther..

[47] Krawczyk A., Stadler S.M., Strzalka-Mrozik B. (2024). Nanomedicines for Dry Eye Syndrome: Targeting Oxidative Stress with Modern Nanomaterial Strategies. Molecules.

[48] Madden L.C., Tomlinson A., Simmons P.A. (2013). Effect of humidity variations in a controlled environment chamber on tear evaporation after dry eye therapy. Eye Contact Lens.

[49] Di Zazzo A., Barabino S., Fasciani R., Aragona P., Giannaccare G., Villani E., Rolando M. (2024). One Soul and Several Faces of Evaporative Dry Eye Disease. J. Clin. Med..

[50] González-García M.J., González-Sáiz A., de la Fuente B., Morilla-Grasa A., Mayo-Iscar A., San-José J., Feijó J., Stern M.E., Calonge M. (2007). Exposure to a controlled adverse environment impairs the ocular surface of subjects with minimally symptomatic dry eye. Investig. Ophthalmol. Vis. Sci..

[51] Georgiev G.A., Yokoi N., Kim F., Bacheva M., Nishiyama M., Hotta T. (2026). Interactions of Mucomimetic Polymers and Meibomian Surface Films upon Exposure to Environmental Stressors. Biomolecules.

[52] Liu C., Madl A.C., Cirera-Salinas D., Kress W., Straube F., Myung D., Fuller G.G. (2021). Mucin-Like Glycoproteins Modulate Interfacial Properties of a Mimetic Ocular Epithelial Surface. Adv. Sci..

[53] Eftimov P., Yokoi N., Kim F., Nishiyama M., Hotta T., Bacheva M., Georgiev G.A. (2026). Ophthalmic Nanoemulsion-Based Mitigation of Environmental Stressors Impact on the Surface Properties of Meibomian Films. Appl. Sci..

[54] Keramatnejad M., DeWolf C. (2023). Impact of Pollutant Ozone on the Biophysical Properties of Tear Film Lipid Layer Model Membranes. Membranes.

[55] Wang H., Jia H., Han J., Zhang Z., Yin X., Mu N., Zhu Y., Li M. (2023). Correlation Between Air Quality Index and Tear Film Lipid Layer Thickness: Comparison Between Patients with Sjogren’s Syndrome and with Meibomian Gland Dysfunction. Curr. Eye Res..

[56] McCulley J., Butovich I., Aronowicz J., Uchiyama E. (2007). Increased Evaporative Rates in Laboratory Testing Conditions Simulating Airplane Cabin Relative Humidity: An Important Factor for Dry Eye Syndrome. Eye Contact Lens Sci. Clin. Pract..

[57] Abusharha A.A., Pearce E.I., Fagehi R. (2016). Effect of Ambient Temperature on the Human Tear Film. Eye Contact Lens.

[58] Abusharha A., Pearce E.I., Afsar T., Razak S. (2023). Protecting Tear-Film Stability under Adverse Environmental Conditions Using a Mucomimetic with a Non-Newtonian Viscosity Agent. Medicina.

[59] Abusharha A.A., Pearce E.I. (2013). The effect of low humidity on the human tear film. Cornea.

[60] Tesón M., González-García M.J., López-Miguel A., Enríquez-de-Salamanca A., Martín-Montañez V., Benito M.J., Mateo M.E., Stern M.E., Calonge M. (2013). Influence of a controlled environment simulating an in-flight airplane cabin on dry eye disease. Investig. Ophthalmol. Vis. Sci..

[61] Ji H., Yang Y., Lu Y., Kong X., Yang G., Liu J., Yang Y., Wang X., Ma X. (2023). Prevalence of dry eye during the COVID-19 pandemic: A systematic review and meta-analysis. PLoS ONE.

[62] (2020). Thermal Environmental Conditions for Human Occupancy.

[63] Su B., Jadresin Milic R., McPherson P., Wu L. (2022). Thermal Performance of School Buildings: Impacts beyond Thermal Comfort. Int. J. Environ. Res. Public Health.

[64] Baeza D.C., Penso J.Z., Menendez D.M., Contreras J.A., Rock S., Galor A., Kumar N. (2025). The Impact of Home Interventions on Dry Eye Disease (DED) Symptoms and Signs in United States Veterans. Int. J. Environ. Res. Public Health.

[65] Jones L., Craig J.P., Markoulli M., Karpecki P., Akpek E.K., Basu S., Bitton E., Chen W., Dhaliwal D.K., Dogru M. (2025). TFOS DEWS III: Management and Therapy. Am. J. Ophthalmol..

[66] Zhang R., Zhang X., Zhang Q., Li Y., Wang Y., Xu J., Cheng Z., Chen H., Yao Y., Sun H. (2024). Heterogeneous Photodegradation Behavior of Liquid Crystal Monomers in Dust: Quantitative Structure-Activity Relationship and Product Identification. Environ. Sci. Technol..

[67] Yang W., Yang K., Pan Y., Wu S., Chen X., Shen L., Zeng Q., Wu J., Lv M., Zhang J. (2023). A literature-derived dataset on risk factors for dry eye disease. Sci. Data.

